# MicroRNAs in Inflammatory Bowel Disease and Its Complications

**DOI:** 10.3390/ijms23158751

**Published:** 2022-08-06

**Authors:** Srikruthi S. Krishnachaitanya, Max Liu, Ken Fujise, Qingjie Li

**Affiliations:** 1 Department of Internal Medicine, University of Texas Medical Branch at Galveston, Galveston, TX 77555, USA; 2 Division of Cardiology, Department of Medicine, University of Washington, Seattle, WA 98109, USA

**Keywords:** Crohn’s disease, ulcerative colitis, non-coding RNA, extraintestinal manifestations, colitis-associated cancer

## Abstract

Inflammatory bowel disease (IBD), classified primarily between Crohn’s disease and ulcerative colitis, is a collection of chronic gastrointestinal inflammatory conditions that cause multiple complications because of systemic alterations in the immune response. One major player is microRNA (miRNA), which is found to be associated with multiple pathways in mediating inflammation, especially those of a chronic nature in IBD, as well as irritable bowel syndrome. Although there have been studies linking miRNA alterations in IBD, even differentiating Crohn’s disease and ulcerative colitis, this review focuses mainly on how miRNAs cause and mechanistically influence the pathologic complications of IBD. In addition to its role in the well-known progression towards colorectal cancer, we also emphasize how miRNA manifests the many extraintestinal complications in IBD such as cardiovascular diseases; neuropsychiatric conditions such as depression and anxiety disorders; and others, including various musculoskeletal, dermatologic, ocular, and hepatobiliary complications. We conclude through a description of its potential use in bettering diagnostics and the future treatment of IBD and its systemic symptoms.

## 1. Introduction

Inflammatory bowel disease (IBD) is a class of chronic inflammatory conditions predominantly arising in the colon and lower gastrointestinal tract that is classified principally between Crohn’s disease (CD) and ulcerative colitis (UC). The incidence of IBD has risen globally in both Western and Eastern countries with suspected attribution in part to globalization and a Western-influenced diet [1,2]. Still, the pathophysiology of IBD remains a field under intense study, detailed as a complex association between genetics and epigenetics, immune system aberrancies, gut dysbiosis, and other environmental factors [3,4]. Among these are microRNAs (miRNAs), a unique class of single-stranded noncoding ribonucleic acids, which alter gene expression via targeting mRNA post-transcriptionally. Many miRNAs are associated with crucial regulation of multiple inflammatory pathways affected in IBD [5].

The class of miRNAs comprises 18-22 nucleotides that repress mRNA translation via base pairing along sequence motifs such as those in the mRNA 3′UTR region [6]. They typically suppress gene expression via cleavage of specific target mRNAs, of which there can be multiple targets per unique miRNA. Thus, a single mRNA sequence is often regulated by multiple miRNAs, which normally assist in fine-tuning the complexity of gene expression. Therefore, any imbalances, especially those in chronic inflammatory disease states such as IBD and cancer, are often, in part, a result of imbalances in miRNA and affected gene expressions.

Though recognized most for its effects on the gastrointestinal tract, IBD is well known for harboring a multitude of complications, including extraintestinal. This largely stems from the chronic and systemic inflammatory state IBD induces via pathways such as NF-κB, STAT3, the NOD2 receptor family, and toll-like receptors (TLRs), which alter regulatory mediators such as cytokines and various miRNAs [5,7]. Perhaps the most well-studied is the strong association between IBD-induced colitis and increased colon cancer risk, arguably the most lethal complication of IBD [8]. However, IBD has also been linked with increased cardiovascular disease and heart failure risk; psychological manifestations, including depression and anxiety; and further complications including uveitis, arthropathy, rash, and cholangitis [9,10]. Overall, miRNAs play major roles in IBD and its chronic inflammatory complications with potential for clinical applications in differentiating CD and UC, indicators for prognosis, and potential suggestions in therapeutic use.

## 2. Dysregulated miRNAs in Patients with UC

UC is one of the main branches of IBD characterized by inflammation of the superficial mucosal and submucosal layers, classically beginning rectally and capable of ascending continuously [11]. Wu et al. first observed the dysregulation of miRNA within IBD and found that miR-192 targets macrophage inflammatory peptide 2α and is downregulated in active UC compared to healthy controls [12]. Since then, multiple miRNAs across various tissue samples have been found to be significantly dysregulated in UC (Table 1). In the human colon, Guz et al. noted five significantly upregulated miRNAs—miRs-21-3p, -31-3p, -125b-1-3p, -146a-3p, and -155-5p—in inflamed UC colonic tissue as opposed to normal-looking adjacent tissue [13]. Using healthy controls, Schaefer et al. found miRs-21 and -31 significantly upregulated in UC colon tissue and additionally noted increased miRs-19a and -101 [14]. Wu et al. conducted an extensive study noting miRNA dysregulation in colon tissue between both active and inactive UC versus healthy patients [12]. Specifically, they found eight miRNAs significantly upregulated in active UC including miRs-16, -23a, -24, -29a, -126, -195, let-7f, and, again, miR-21. In addition, three miRNAs, namely miRs-192, -375, and -422b, were significantly downregulated in active UC. Interestingly, differential miRNA expression exists between active and inactive UC. For example, although miRs-375 and -422b are downregulated in active UC colon, they are upregulated in inactive UC colon. An additional six miRNAs in colon biopsies can also be differentiated between active and inactive UC: miRs-16, -21, -24, -126, -203, and -200b.

Peripheral blood serves as another sample type in which miRNAs are dysregulated compared to healthy individuals. Schaefer et al. noted six significantly upregulated miRNAs in the peripheral blood of UC versus healthy patients: miRs-19a, -101, -142-5p, -223, -375, and -494 [14]. Again, it is important to distinguish miRNA expression variability depending on sample type. Although miR-375 is downregulated in active UC colon biopsies, it is upregulated in the peripheral blood of UC patients [12,14]. Wu et al. additionally found twelve miRNAs significantly upregulated in active UC peripheral blood including miRs-28-5p, -30e, -103-2, -151-5p, -199a-5p, -215, -340, -362-3p, -374b, -532-3p, -638, and miRplus-E1271 [15]. Again, there may be utility in distinguishing miRNAs in the peripheral blood of active versus inactive UC patients, specifically with the five miRs-28-5p, -151-5p, -199a-5p, -340, and miRplus-E1271 upregulated only in active UC. Downregulated miRNAs in UC peripheral blood interestingly included miRs-21, -31, -146a, and -505 [14,15]. Again, the first three listed are a direct contrast to colon tissue upregulation, emphasizing the variation among sample types, though some studies did note elevated serum miR-21 in more severe active disease [13,14,19]. Using next-generation sequencing, we identified 20 plasma exosomal miRNAs differentially expressed in patients with UC vs. healthy control subjects, 13 of which (miRs-29b-3p, 96-Sp, 624-Sp, 186-Sp, 1,303, 4,487, 20b-Sp, 503-Sp, 363-3p, 194-Sp, 548au-5p, 942-3p, and 218-Sp) were upregulated and 7 (miRs-31- Sp, 3130-3p, 7851-3p, 4433b-3p, 485-3p, 202-Sp, and 224-Sp) downregulated [21].

miRNA dysregulation has also been found in stool samples of UC patients. Verdier et al. noted significantly elevated fecal miRs-223 and -1246 in active UC patients compared to healthy controls [18]. Schönauen et al. similarly found miR-223 upregulated by over 67-fold in active UC patient feces and also significantly increased miRs-16 and -155 [19]. Ahmed et al. observed upregulated miRs-21, -126, -203, and, again -16, as well as downregulated miRs-192 and -320 [20]. In addition to fecal miRNAs, salivary miRNAs provide yet another non-invasive method of discernment. Schaefer et al. noted significantly upregulated miRs-21, -31, and -142-3p with downregulated miR-142-5p in UC saliva samples compared to normal controls [14]. Clearly, miRNAs have much potential to help discern patients with UC outside of only colon biopsies—in peripheral blood, stool, and saliva samples.

Briefly, we summarize the pathogenesis that may be involved with several key miRNAs. It has been found that miRs-21 and -155 are associated with regulating the activity of various TLRs and potentially have the capacity of binding TLRs [22], which play a major role in proper interaction with gut microbiota and immune activation via NF-κB, among others [23,24]. miR-21 upregulation in IBD encourages T cell activation in UC remission patients and may potentially reduce tumor suppressor PDCD4 expression in CD3+ T cells, promoting inflammatory progression and eventual cell proliferation towards cancer [25]. Upregulated levels of miRs-31 and -155 in UC have been shown to regulate increased IL-13 levels by downregulating expression of IL13Rα1, the primary receptor subunit for IL-13 [26]. Increased IL-13 levels in Th2-mediated UC are involved in epithelial barrier dysfunction via altered claudin-2 expression in tight junctions and an increased rate of apoptosis [27,28]. E-cadherin expression is also downregulated by miR-155, further decreasing mucosal stability in UC and increasing metastatic cancer risk with future progression [13]. Finally, miR-375 downregulation in colon biopsies is directly associated with reduced targeted regulation of CTGF-EGFR with subsequent upregulated tissue growth contributing towards cancer progression [29].

## 3. Dysregulated miRNAs in Patients with CD

In contrast to UC, CD classically involves transmural inflammation, which can impact the entire GI tract, though not necessarily continuously, with skip lesions often affecting the terminal ileum and colon [30,31]. It was found that miRs-21-3p, -31-3p, -146a-3p, and -155-5p were significantly overexpressed in inflamed CD ileal and colonic tissue compared to normal-looking adjacent tissue [13]. Schaefer et al. supported the dysregulated increase in miRs-31 and -146a in CD patients, but additionally noted miR-101 upregulation [14]. miRNAs are variably expressed depending on regional location [32]. Wu et al. determined three miRNAs were significantly upregulated in sigmoid colon biopsies of Crohn’s colitis patients versus healthy adults—miRs-23b, -106a, and -191. Four miRNAs were upregulated in Crohn’s ileitis patients including miRs-16, -223, -594, and, as supported, miR-21. Downregulated miRNAs in CD patients comprised miRs-19b, -375, and -629 [14,32]. Of note, miR-29 was also downregulated in CD mucosa along stricture regions [33].

In the peripheral blood of CD patients, Schaefer et al. noted only significantly upregulated miRs-101 and -375 [14]. Another study from Wu et al. revealed significant upregulation of the five miRs-199a-5p, -340, -362-3p, -532-3p, and miRplus-E1271 in the peripheral blood of active CD patients compared to healthy controls [15]. However, similar to distinguishing active from inactive UC, only miR-340 was significantly upregulated in inactive CD peripheral blood, and therefore can be differentiated from the active form by the presence or absence of the other four miRNAs. Nijhuis et al. noted significantly upregulated sera miR-29 [33]. Six downregulated miRNAs in the peripheral blood of CD patients included miRs-21, -31, -146a, -149, -155, and miRplus-F1065 [14,15]. Again, miR-21 serum elevation in other studies is somewhat dependent on active disease [19].

For fecal CD samples, Wohnhaas et al. distinguished nine significantly upregulated miRNAs consisting of miRs-15a-5p, -16-5p, -24-3p, -27a-3p, -128-3p, -142-5p, -223-3p, -223-5p, and -3074-5p compared to healthy controls [34]. Another study by Schönauen et al. additionally noted significantly upregulated fecal miRs-155 and -223 alongside miR-16 [19]. Wohnhaas et al. also recognized significant downregulation of eight fecal miRNAs including miRs-10a-5p, -10b-5p, -141-3p, -192-5p, -200a-3p, -375, -378-3p, and let-7g-5p [34]. Similar to UC, salivary samples in CD patients may be used, as miRs-26a and -101 were found to be significantly upregulated [14]. Dysregulated miRNAs in patients with CD are summarized in Table 2.

miRNAs play major roles in CD pathogenesis and inflammatory regulation, comparable to UC. It was found that miR-192 served to suppress NOD2 receptor activity in colonocytes, thus reducing inflammatory activation [35]. However, miR-192 downregulation may contribute toward NOD2 overactivation via muramyl dipeptide and, thus, influence CD progression. Another study found that NOD2 signaling raised miR-29 levels, which helped modulate IL-12p40 expression, a component of the cytokines IL-12 and IL-23, reducing Th1 and Th17 stimulation [36]. However, in CD, NOD2 mutations resulted in reduced miR-29, increased inflammatory response, and worsened colitis. Additionally, miR-29b downregulation is related to increased TGF-β signaling, which promotes profibrotic activity and stricture formation in CD [33]. Downregulation of miR-200 is associated with epithelial to mesenchymal transition (EMT) dysregulation via loss of E-cadherin in CD [37], similar to the actions of miRs-31 and -155 [13].

## 4. miRNAs in the Pathogenesis of Complications Associated with IBD

**Figure 1:**
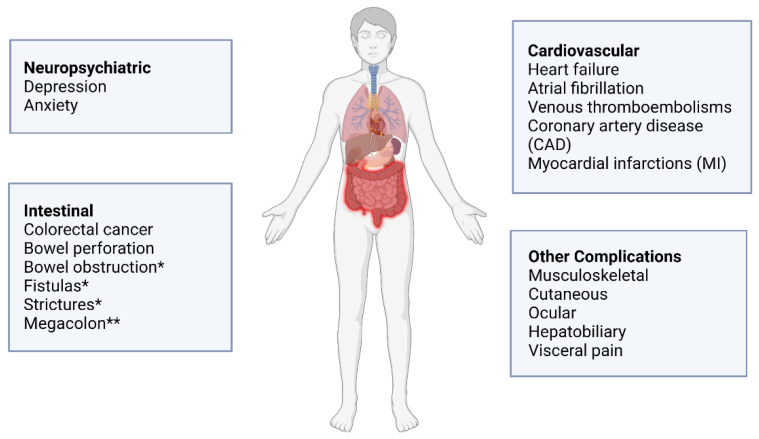
Intestinal and extraintestinal complications of IBD. * Mainly associated with Crohn’s disease; ** mainly associated with ulcerative colitis. Created with Biorender.com.

Both CD and UC are systemic chronic inflammatory diseases, which may lead to both intestinal and extraintestinal complications. General complications in IBD include colitis-associated cancer, cardiovascular diseases, neuropsychiatric illnesses, and other systemic complications (Figure 1). For many of these IBD-associated complications, miRNAs are implicated in many facets, including the potential modulation of its onset, progression, and prognosis. The ever-emerging evidence that miRNAs are important regulators in disease processes increases their viability of becoming diagnostic indicators and therapeutic targets.

### 4.1. miRNAs in Colitis-Associated Colorectal Cancer

Colitis-associated colorectal cancer (CAC) is a major complication that accounts for 10–15% of deaths among IBD patients and favors a worse prognosis compared to spontaneous colorectal cancer (CRC) [38]. While surgical procedures such as mucosectomy and proctocolectomy lower the risk of CAC, these measures do not completely ameliorate the risk, with one-sixth of UC patients suffering mortality from CAC [8,39]. The chronic inflammation in UC and CD increases the risk of CAC as dysregulated cytokines, miRNAs, transcription factors, inflammatory mediators, and gut dysbiosis participate in the process of transforming chronic colonic injury into neoplasia [40,41,42,43]. Numerous studies have shown that miRNAs may significantly influence cancer tumorigenesis, proliferation, invasion, and metastasis [41,44,45]. Increased expression of TLR4 is a distinctive characteristic of CAC that co-occurs with miR-155 upregulation alongside downregulation of suppressor of cytokine signaling 1 (SOCS1) and Src homology 2 domain-containing inositol-5′-phosphatase 1 in SW480 and HCT116 cancer cells [42,46]. This increased activation leads to constitutive STAT3 activation. Upregulated TLR4 signaling alongside upregulated miR-9, miR-25, miR-92a, and miR-301A may ultimately induce epithelial to mesenchymal transition (EMT) by targeting E-cadherin, a cell adhesion protein, to promote tumor invasion and metastasis [38,46,47].

miR-19a contributes to tumor initiation by activating NF-κB signaling through TNF-α-induced protein 3 [48]. miR-20a from stromal cells directly represses the 3′UTR of CXCL8, an inflammatory chemokine secreted from interstitial fibroblasts, and its dysregulation was postulated to modulate tumor genesis, but not influence tumor outcome [49]. Butin-Israeli et al. found that polymorphonuclear neutrophils (PMNs) may be involved in inducing genomic instability through double-strand breaks in colonic inflammation and potentially neoplasia [50]. PMN infiltration of colonic mucosa was followed by the release of miR-23a and miR-155, which may increase the collapse of replication forks through lamin-B1 downregulation and interfere with homologous recombination through targeting regulator RAD51, which also assists in the repair of DNA double-strand breaks.

**Figure 2:**
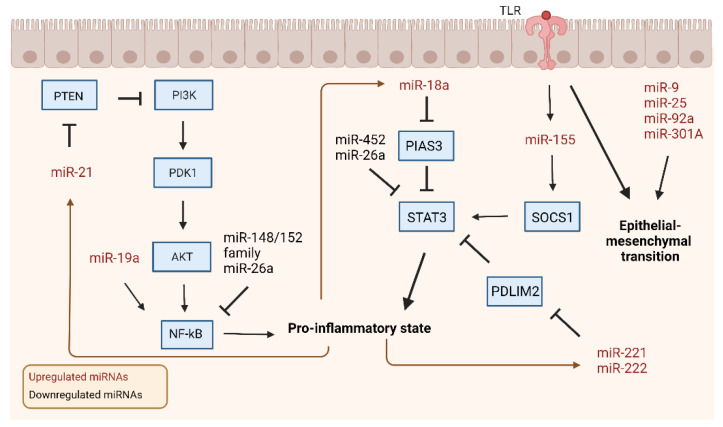
miRNAs involved in CAC-associated canonical signaling pathways. In the development of CAC, major signaling pathways involve the upregulation and downregulation of several miRNAs. miRNA upregulation leads to the increased expression of proinflammatory cytokines, morphological changes, and increased epithelial barrier permeability. Feedback contributes to CAC progression by further exacerbating these changes. After activation of the proinflammatory state, these associated changes go back and stimulate miRNAs to create positive feedback mechanisms. The loss of major downregulated miRNAs leads to increased proinflammatory pathway activation, leading to the compounding effects of inflammation on accelerating tumor development. Black arrow: activation. Brown arrow: positive feedback. Created with Biorender.com.

Feedback loops involving miRNAs are implicated in the fast progression of CAC as compared to spontaneous CRC (Figure 2). miR-21 is a major upregulated miRNA that plays a variety of roles in tumor genesis and development. Lai et al. illuminated how miR-21 affects the major pathways of PI3K/AKT, IL-6/JAK/STAT3, and PDCD4/NF-κB/TNF-α during carcinogenesis within a zebrafish model [45]. miR-21 is activated through gut dysbiosis and targets tumor suppressors PDCD4, BTG2, and TPM1, along with modulating the PI3K/AKT pathway by repressing PTEN to activate ERK and AKT, which induces the downstream NF-κB pathway to release inflammatory cytokines TNF-α, IL-6, and IL-1β [45,51]. IL-6 is then able to use the JAK signaling pathway to activate STAT3, which enables it to create a positive feedback loop by activating the promoter region of miR-21. Upregulation of miR-18a in CRC/CAC colon tissues was also able to induce a positive feedback loop of inflammation through downregulating PIAS3 to increase NF-κB and STAT3 activation, which, in turn, increases miR-18a expression [52]. miR-222 and miR-221 undergo a similar feedback loop mechanism through targeting PDZ and LIM domain 2 to increase the stability of RELA and STAT3 proteins by directly binding to RELA to increase its stability to further activate the NF-κB/STAT3 pathways [53].

A few miRNAs that help control the inflammatory response include miR-148, miR-143, miR-145, miR-452, and miR-26a. miR-148 targets IKKα, IKKβ, IL1R1, GP130, and TNFR2 to decrease NF-κB pathway activation and was downregulated through promoter hypermethylation in dextran sodium sulfate- (DSS)/azoxymethane- (AOM) treated mice [54]. Members of the miR-148/152 family in the DSS-/AOM-treated mice suppress the TNF-α/NF-κB signaling pathway by decreasing matrix metalloproteinase 10 (MMP10) and MMP13 expression [55]. miR-143 and miR-145 were also found to inhibit tumor development and progression and suppress A Disintegrin and Metalloproteinase17, a known tumor development promoter, in DSS-/AOM-treated mice [56]. Lamichhane et al. found that miR-452, a miRNA with differential expression patterns reported based on cancer type, directly targets IL20RA in CRC cell lines to decrease inflammatory protein regulators such as JAK1, STAT1, and STAT3, but interestingly, the inhibition of miR-452 did not rescue STAT1 levels, indicating that miR-452 does not directly modulate it [57]. miR-26a overexpression in myeloid cells was able to suppress IL-6 production, as well as NF-κB and STAT3 activation in macrophages to ameliorate DSS-induced colitis, though the exact target of miR-26a remains unknown [58]. Furthermore, while Chen et al. reported that IL-6 did not have a transcript for direct miR-26a binding, possible explanations may be the use of different cell lines in the experiment or the presence of divergent modulatory mechanisms within different cancers [59]. Because current research is uncovering a wealth of important roles that miRNA plays within CAC, which are summarized in Table 3, future directions for research may focus on therapeutic and diagnostic uses of miRNA.

### 4.2. miRNAs in Cardiovascular Complications of IBD

**Figure 3:**
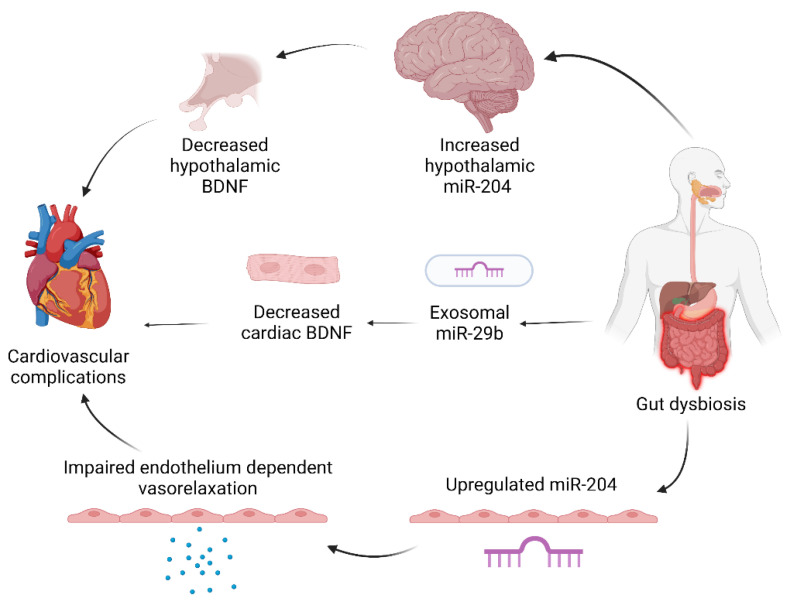
IBD affects the heart through multiple mechanisms. Gut dysbiosis can contribute to cardiovascular complications through affecting indirect neurogenic signaling, directly sending exosomes to modulate cardiac regulators, and impairing vasorelaxation through circulating inflammatory markers. Created with Biorender.

IBD is associated with an increased incidence of heart failure and concurrent hospitalization, atrial fibrillation, venous/arterial thrombosis, coronary artery disease, and myocardial infarction (MI) [61]. There is emerging evidence that the systemic inflammatory processes of IBD may contribute to the pathogenesis of cardiovascular diseases through increasing inflammatory mediators such as reactive oxygen species, C-reactive protein (CRP), and pro-inflammatory cytokines [61,62]. miRNAs are being implicated as signaling molecules in pathogenic mechanisms, as well as potential players in gut–heart crosstalk (Figure 3).

Vikram et al. reported how gut microbiota can promote atherosclerosis through remotely regulating miR-204 in mouse aorta endothelium [63]. Upregulated miR-204 decreases Sirt1, a class III histone deacetylase that acts on endothelial nitric oxide synthase, which leads to decreased endothelial nitric oxide to ultimately impair endothelium-dependent vasorelaxation. Impaired vasorelaxation is a marker for early atherosclerosis, and it is proposed that phosphorylated STAT3, a repressor of miR-204, is a potential gut–heart signaling target as it was subsequently downregulated in mice with high-fat diets, though it is potentially not the only target, as Sirt1 knockout did not completely ameliorate miR-204 upregulation. Gut dysbiosis may also impact hypothalamic miR-204, as studies show an association between decreased hypothalamic miR-204 and increased hypothalamic brain-derived naturopathic factor (BDNF) [64]. Increased BDNF in the hypothalamic paraventricular nucleus is associated with arrhythmia, hypertension, and sympathetic activity, which indicates that gut dysbiosis may alter cardiovascular activity through indirect neurogenic pathways.

Exosomes carrying miRNA are another potential route in how colitis remotely affects different organs [21]. We found that inflammatory mediators TNF-α, IL-1β, and H_2_O_2_ in colonic epithelial cells can increase exosomal miR-29b, which modulates several genes coding for cardiac growth and homeostatic factors including BDNF and MYLPF to ultimately facilitate cardiac remodeling. We also found that chronic colitis increases several cardiac miRNAs, including miR-155, which targets BDNF, a neurotrophin that displays diverse protective effects on cardiac function such as endothelial cell survival, post-MI ischemic tissue neovascularization, antioxidant function, and angiogenesis [65,66]. While TNF-α inhibitors are a mainstay treatment for IBD, there may be therapeutic potential for inflammatory cytokine blockers such as the anti-IL-1β antibody to decrease miRNA mediators and ameliorate cardiac remodeling, but more research will need to be conducted on the mechanisms involved. It is postulated that the epithelial barrier dysfunction induced by chronic colitis creates a leaky gut, which can transport the endotoxins, cytokines, proteases, miRNAs, and other inflammatory stimuli from gut dysbiosis through the bloodstream and activate systemic inflammatory processes, but future research will need to elucidate the precise mechanisms.

### 4.3. miRNAs in Mental Health Complications of IBD

Neuropsychiatric disorders such as anxiety and depression are closely comorbid with IBD and gut dysbiosis [67]. Multiple studies have shown that gut microbiome/brain dysregulation creates bidirectional alterations in gut mechanics, stress modulation, and cognitive processing [68,69,70]. miRNAs are implicated in proper gut microbiome maintenance, as the elimination of the DICER processing enzyme creates alterations in intestinal epithelial integrity and gut microbiota diversity [69]. The gut and brain also display remotely mediated crosstalk, as Jang et al. showed that fecal microbiota transplant (FMT) from patients with IBD with or without depression significantly increased anxiety-like behaviors within mice, and mice transplanted with FMTs from patients with IBD and depression showed depression-like behaviors as well [71]. There is also evidence that IBD can facilitate organizational changes within neurological structures, which could be mediated by miRNAs [64].

One neurological mediator that miRNAs modulate so far is BDNF. BDNF is an important neurotrophic factor that affects several neurological functions such as cellular proliferation, synaptic functioning, and neuronal survival [72,73]. Huan et al. found that miR-155 increased, while BDNF and lncRNA MIR155HG decreased within the hippocampus in mice with chronic unpredictable mild stress (CUMS) and that miR-155 suppresses BDNF expression through direct binding [74]. MIR155HG was also shown to repress miR-155, and MIR155HG overexpression ameliorates depression-like behaviors within mice. While this may be a future therapeutic target, further research needs to be performed on miR-155 expression in neuropsychiatric disorders within a colitis model. Antagomirs have been able to reduce depression-like behavior, and Yang et al. showed that an miR-124 antagomir was able to rescue reduced BDNF and CREB1 in rat hippocampi and subsequently increase norepinephrine, dopamine, and serotonin levels in the mice undergoing CUMS [75]. Future directions of IBD research may focus on illuminating how miRNAs are involved in neuropsychiatric manifestations and their potential in ameliorating IBD and its systemic complications.

### 4.4. miRNAs in Other Complications of IBD

Extraintestinal manifestations (EIMs) in IBD are extremely common with estimations that approximately 5–50% of IBD patients experience at least one EIM, which may significantly impact a patient’s prognosis and quality of life (Figure 1) [76]. The most common EIMs include musculoskeletal, cutaneous, ocular, hepatobiliary, and visceral pain [77,78]. Knowledge about miRNA involvement in EIMs is limited, which highlights a growing need for illuminating the mechanisms behind the systemic pathogenesis of IBD. Interestingly, 75% of patients with primary sclerosing cholangitis (PSC) are diagnosed with IBD, indicating that the underlying pathological mechanisms between them may intersect, leading to their strong comorbid relationship [76]. It has also been shown that this disease combination carries a significant risk for CAC and displays a significant increase of miR-155 and TLR4 expression, accompanied by a significant downregulation of SOCS1 protein in PSC peripheral blood mononuclear cells [46]. miR-155 may be potentially involved in the pathogenesis of PSC through downregulation of SOCS1, but further research will be needed to clarify the exact mechanism. Visceral pain is another complication that can severely impact quality of life for affected IBD patients. Lu et al. found that miR-146a-5p directly targets the 3′UTR of CCL8, which prevents CCL8 from activating receptor CCR5 for visceral hyperalgesia in a model of *trinitrobenzene-sulfonic-acid*-induced colitis [77]. Emerging evidence is starting to recognize the role of miRNAs as mediators in the systemic manifestations of IBD, and future research will be needed to fully elucidate the role of miRNAs in extraintestinal pathogenic mechanisms.

## 5. miRNAs in IBD-Associated Diagnostics

IBD is currently diagnosed through a multitude of different assessments including clinical history, radiology, endoscopy, colonoscopy, and histology [79,80]. However, these invasive procedures carry risks such as perforation and bleeding. Diagnostic challenges remain in differentiating between UC and CD when lesions are solely limited to the colon and in differentiating between IBD and irritable bowel syndrome (IBS) [80]. Endoscopy represents the main method of differentiation between organic IBD, and more functional IBS disorder though inflammatory markers such as TNF-α [81] and calprotectin [82,83] has also been used. miRNAs are found to be stable in peripheral blood, saliva, and feces and have been suggested as diagnostic biomarkers of IBD [80]. There is also research indicating that miRNAs can serve as sensitive and specific biomarkers for disease onset, prognosis, and remission [79,80].

### 5.1. Differentiating UC and CD with miRNAs

**Figure 4:**
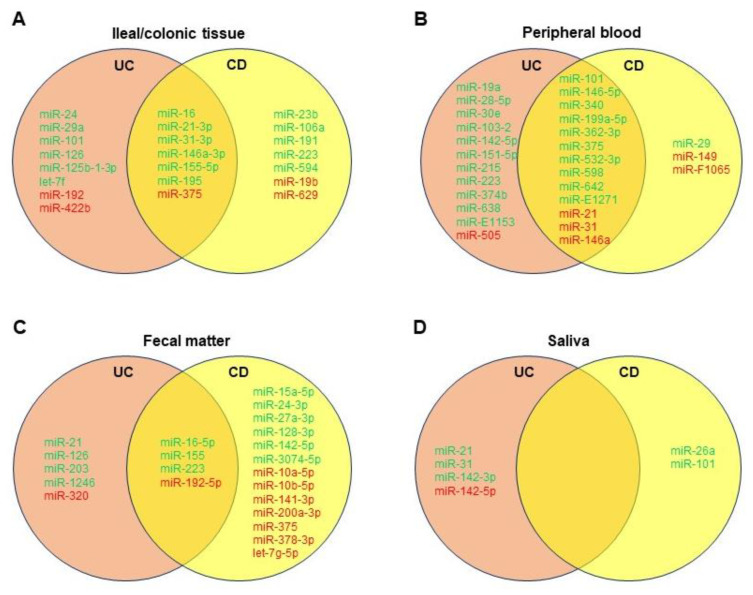
Venn diagrams showing significantly dysregulated miRNAs in ileal/colonic tissue (**A**), peripheral blood (**B**), fecal matter (**C**), and saliva (**D**) of patients with ulcerative colitis (UC) or Crohn’s disease (CD). Green denotes upregulated miRNAs, and red denotes downregulated miRNAs compared to healthy controls.

While distinguishing between UC and CD remains a diagnostic challenge, newer biomarkers such as human alpha defensin 5 and miRNAs may aid in diagnosis, especially in histologically indeterminate scenarios [84]. Forming a miRNA panel may help serve as a potential tool to distinguish between CD and UC and evaluate complications of IBD, as discussed. In addition, utilizing differential miRNAs for diagnosis is much less invasive than classic endoscopy, and yet as specific [14,15]. Based on the literature, we created Venn diagrams showing common differentially dysregulated miRNAs in the ileal/colonic tissue (Figure 4A), peripheral blood (Figure 4B), feces (Figure 4C), and saliva (Figure 4D) of patients with UC or CD. Albeit that further validation is needed, different miRNA panels could be established and utilized for differential diagnosis of UC and CD.

One study from Wu et al. noted eight confirmed miRNAs in peripheral blood that were distinguishable between active UC and active CD: miRs-28-5p, -103-2, -149, -151-5p, -340, -532-3p, and miR-plus-E1153 were all significantly elevated in active UC when compared to active CD, and miR-505 was significantly decreased in active UC versus active CD [15]. The study also noted these miRNA “signatures” were more homogenous in the peripheral blood samples of active disease patients as opposed to inactive remission patients. Another study determined after screening assays that miRs-598 and -642 were significantly upregulated in the UC patients’ plasma in comparison to CD patients’, though both were significantly elevated in UC and CD compared to healthy controls [16]. Furthermore, miRs-16, -21, and, in particular, -223 were more prominently increased in active CD sera versus that of active UC [19].

In fecal matter, miRs-223 and -1246 were significantly upregulated in UC individuals with fecal calprotectin > 250 mg/kg compared to CD individuals with fecal calprotectin < 250 mg/kg [18]. Studies from Ahmed et al. and Wohnhaas et al. seem to suggest upregulation of miRs-21, -126, and -203 and downregulation of miR-320 may distinguish UC from CD [20,34]. In general, it seems proinflammatory miRNA expression is higher-fold in feces of UC patients compared to CD patients, though this depends on other variables as well, including disease activity [19]. Salivary miRNAs are still quite novel, though from our review, it seems miRs-26a and -101 may be more prominent in CD patients [14].

Even with colonic tissue biopsies, a more specific and complementary miRNA panel may better support histology if colonoscopy is to be undertaken. For example, although miR-29a is significantly upregulated in UC colon samples, miR-29 family members in CD colon tissue are not, with downregulation even occurring along strictures via TGF-β-mediated fibrosis [33]. Furthermore, miR-125b-1-3p was determined by Schaefer et al. as only significantly upregulated in UC colon tissue [14]. Other miRNAs to consider may include miRs-31, -106a, -146a, -192, and -375, though further research with larger sample sizes with attention toward disease activity may improve the knowledge of various miRNAs involved in either UC or CD.

One diagnostic difficulty in IBD is the differentiation between clinical and endoscopic remission, where clinical remission is the absence of symptoms, but endoscopic remission is the absence of detectable mucosal lesions and is associated with better clinical prognosis [79]. miR-320a was found to be increased in the peripheral blood of CD patients with active disease flares compared to those with quiescent disease; was directly correlated with disease severity; was associated with endoscopic disease activity in the setting of mild or absent clinical symptoms; was increased in CD patients with extensive intestinal involvement; and was elevated compared to patients with *C. difficile*-associated colitis [79,85]. While miR-320a is a candidate for being a diagnostic biomarker for detecting differentiation of active colitis, Cordes et al. showed that it does not correlate with histological colitis activity, and larger cohort studies are needed to fully assess the diagnostic value [17]. For serum miR-146b-5p, CD and UC patients had levels 2.87-fold higher and 2.72-fold higher than age- and gender-matched healthy controls. Chen et al. found that miR-146-5p had potential diagnostic value as its expression had similar sensitivity to CRP, a marker that is associated with disease activity in UC/CD, and displayed increased specificity compared to CRP (92.31% vs. 46.15%).

### 5.2. Differentiating IBS and IBD with miRNAs

miRNAs may help differentiate IBS from IBD and its complications. One study noted significant upregulation of miRs-23a, -375, and -422b in IBS colonic tissue compared to healthy controls [12]. Zhou et al. found upregulated miRs-29a and -29b in the small intestinal and colonic tissue of IBS-D patients [86]. In colonic mucosa, miRs-219a-5p and -338-3p were found downregulated in IBS patients [87]. miR-375 may also be useful for IBS/IBD differentiability exhibiting downregulation in IBD colonic tissue, in contrast to IBS upregulation. For CD specifically, miR-29b may distinguish CD versus IBS with downregulation and upregulation, respectively.

In serum studies, proinflammatory miRs-23a and -181b are upregulated in IBS patients compared to healthy controls [88]. Moreover, miRs-150 and -342-3p were also found upregulated in IBS patients [89]. Conversely, serum miR-199b levels were downregulated [90]. Another study identified miRs-21 and -92a upregulated in UC compared to IBS patients [91]. Just like miRNAs can distinguish UC and CD, more research may help solidify differential miRNAs in IBS patients with consideration for specific disease variation amongst IBS-C, IBS-D, and IBS-M.

### 5.3. Challenges and Future Indications for miRNA-Based Diagnostics

Some challenges that miRNAs face as a diagnostic profiling tool is the challenge of normalizing peripheral fluid data to obtain standard cut-off values, low sensitivities and specificities in current detection methods, and creating point-of-care assays to decrease diagnostic latency [80,92,93]. While miRNAs may prove to be useful diagnostic prognosticators, more information is needed with regard to tissue/sera normalization across a cohort with a wide variety of patient backgrounds to increase validity. Furthermore, because of the multiplicity of roles that a single miRNA is involved in, it may be useful to try using a panel-based approach, as discussed above, for future research and diagnostic purposes. Current detection methods of miRNAs include traditional methods such as Northern blotting, microarrays, and RT-qPCR, while newer detection methods include nanomaterial-based miRNA detection, nucleic acid amplification techniques, rolling circle amplification, fluorescent in situ hybridization, strand displacement amplification, loop-mediated isothermal amplification, and enzyme-free amplification [92]. While traditional RNA detection methods are predominantly employed, these methods suffer from limitations such as low specificity and sensitivity and applications requiring technical expertise and can be time consuming. Newer detection techniques display increased sensitivity and specificity and increased discriminatory ability and can potentially be more cost-effective. These technologies may play a role in point-of-care settings as microfluidic chip- and electrochemical-based systems augment these newer detection routes to become more portable than current diagnostics modalities [93]. However, these newer techniques still display limitations such as cost and complexity, as well as require further sensitivity and specificity validation. The future of miRNA detection will require a widespread validation of newer techniques, as well as the employment of a combination of existing techniques to utilize miRNA diagnostics in a cost-effective, widespread manner.

## 6. miRNAs in IBD-Associated Therapeutics

Current therapeutic interventions for IBD include aminosalicylates, thiopurines, corticosteroids, and biologics [94]. Therapeutic use of miRNAs is being considered within research as a novel treatment agent due to the ability of miRNAs to simultaneously modulate multiple gene targets [95]. Currently, there are two main strategies of treatment: miRNA antagonists and miRNA mimics.

miRNA antagonists are composed of antisense oligonucleotides that preferentially inhibit miRNA’s “seed regions” to induce silencing of downstream pathways with antagomirs including functional groups for nuclease degradation [96,97]. Suri et al. also reported that a variety of interventions that inhibited miR-29a, miR-26b, miR-233, miR-19a, miR-146-5p, and miR-122a were able to restore epithelial barrier integrity and alleviate symptoms of IBD [97]. miR-214 may serve as a potential therapeutic target, as it is overexpressed in UC and CAC colonic tissue and it decreases PTEN and PDLIM2 to activate the NF-κB and AKT signaling pathways and amplify the inflammatory response [98]. miRNA antagomirs may also augment existing clinical therapies with let-7a inhibition increasing Fas and Fas ligand in bone-marrow-derived mesenchymal stem cells to induce T cell migration and apoptosis to ultimately maximize mesenchymal stem cell cytotherapy benefits [99]. miRNAs may be a candidate for future therapeutic usage, and currently, Miravirsen is an antimir used for treating the hepatitis C virus (HCV) through targeting miR-122-HCV inhibition and is currently in phase II clinical drug trials [94]. However, antagomir therapy faces hurdles including a lack of specificity for target cells, potential hepatotoxicity, and potential side effects due to vast miRNA gene modulation [94,97]. While liposomal packaging and serum exosomal therapy are suggested as potential solutions, experiments must be replicated in larger cohorts and the variety of animal models increased to ascertain true clinical viability.

Agomirs or miRNA mimics are useful for rescuing the downregulation of miRNA in certain pathological states. However, agomir therapy faces additional hurdles such as a larger dosage requirement and the challenge of incorporating the miRNA into the fully functional RISC complex [94]. miRNA mimics are also being used as burgeoning treatments in oncology with MRX34, a liposomal encapsulated miR-34a mimic, currently in phase I clinical drug trials.

miRNAs may also have a role in predicting individual drug therapy response with a study finding significant downregulation of miR-16-2-3p, miR-30e-3p, miR-32-5p, miR-642a-5p, miR-150-5p, and miR-224-5p within a cohort displaying resistance to glucocorticoid therapy [100]. These results had specificities of 97.30%, 89.20%, 59.50%, 73.00%, 97.30%, and 97.30%, respectively, and displayed sensitivities of 74.40%, 84.60%, 97.40%, 92.30%, 66.70%, and 89.70%. Interestingly, phosphoinositide-3-kinase adaptor protein 1, which affects histone deacetylase activity, was found to be a common gene target for miR-30e-3p, miR-32-5p, and miR-16-2-3-3p and may activate the PI3K/AKT signaling pathway to induce glucocorticoid resistance. Limitations of this study include a small sample size of 18 individuals and a homogenous population. Replication of this study with larger heterogenous cohorts would increase the clinical validity and further strengthen these exciting results.

## 7. Conclusions

Wu et al.’s pioneering study of comparatively looking at miRNAs in UC opened the field of looking at miRNA expression within IBD, including its pathogenic mechanisms and associated complications, and may potentially play a large role in future diagnostic and therapeutic directions [12]. Their ability to post-transcriptionally alter the expression of a multitude of genes makes them attractive targets for drug manipulation, and their stability and presence in a multiplicity of peripheral tissues gives them promise to be non-invasive diagnostic indicators. While there are still many questions within the realm of miRNAs in IBD, this dynamic and rapidly expanding field continues to produce exciting results and provide clinical potential for the future.

## Figures and Tables

**Table 1:** miRNAs dysregulated in UC across various tissue samples.

Scheme 16.	miRNAs Upregulated	miRNAs Downregulated	References
Ileal/colonic tissue	miRs-16, -19a, -21(-3p), -23a, -24, -29a, -31(-3p), -101, -125b-1-3p, -126, -146a-3p, -155(-5p), -195, let-7f	miRs-192, -375, -422b	[12,13,14]
Peripheral blood	miRs-19a, -28-5p, -30e, -101, -103-2, -142-5p, -146-5p, -146b-5p, -151-5p, -199a-5p, -215, -223, -340, -362-3p, -374b, -375, -494, -532-3p, -598, -638, -642, miRplus-E1271	miRs-21, -31, -146a, -505	[14,15,16,17]
Fecal matter	miRs-16, -21, -126, -155, -203, -223, -1246	miRs-192, -320	[18,19,20]
Saliva	miRs-21, -31, -142-3p	miR-142-5p	[14]

Sample types listed include ileum/colon tissue biopsies, peripheral blood, fecal matter, and saliva from UC patient studies compared to healthy sample controls.

**Table 2:** miRNAs dysregulated in CD across various tissue samples.

	miRNAs Upregulated	miRNAs Downregulated	References
Ileal/colonic tissue	miRs-16, -21(-3p), -23b, -31(-3p), -106a, -146a-3p, -155(-5p), -191, -195, -223, -594	miRs-19b, -375, -629	[13,14,32,33]
Peripheral blood	miRs-29, 101, 146-5p, -146b-5p -199a-5p, -340, -362-3p, -375, -532-3p, -598, -642, miRplus-E1271	miRs-21, -31, -146a, -149, miRplus-F1065	[14,15,16,17,33]
Fecal matter	miRs-15a-5p, -16-5p, -24-3p, -27a-3p, -128-3p, -142-5p, -155, -223(-3p and -5p), -3074-5p	miRs-10a-5p, -10b-5p, -141-3p, -192-5p, -200a-3p, -375, -378-3p, let-7g-5p	[19,34]
Saliva	miRs-26a, -101		[14]

Sample types listed include intestinal biopsies from both the ileum and colon, peripheral blood samples, fecal matter, and saliva samples from CD patients (vs. healthy sample controls).

**Table 3:** miRNAs significantly upregulated or downregulated within CAC and their respective roles.

miRNA	Regulation	Role in CAC	Reference
miR-155	Upregulated	Invasion, transformation, tumor genesis	[42,46,50]
miR-18a	Upregulated	Tumor development	[52]
miR-19a	Upregulated	Tumor genesis	[49]
miR-21	Upregulated	Tumor genesis, invasion, development	[45,60]
miR-221	Upregulated	Tumor development	[53]
miR-222	Upregulated	Tumor development	[53]
miR-23a	Upregulated	Tumor genesis	[50]
miR-25	Upregulated	EMT, invasion	[38,46]
miR-301A	Upregulated	EMT, invasion	[38,46,47]
miR-9	Upregulated	EMT	[38,46]
miR-92a	Upregulated	EMT, invasion	[38,46]
miR-143	Downregulated	Inhibits tumor development, progression	[55,56]
miR-145	Downregulated	Inhibits tumor development, progression	[55,56]
miR-148	Downregulated	Inhibits tumor development, progression	[55]
miR-26a	Downregulated	Suppresses inflammatory cytokines	[58,59]
miR-452	Downregulated	Inhibits inflammatory protein regulators	[57]

## Data Availability

Not applicable.

## References

[1] Windsor J.W., Kaplan G.G. (2019). Evolving Epidemiology of IBD. Curr. Gastroenterol. Rep..

[2] Kuenzig M.E., Fung S.G., Marderfeld L., Mak J.W.Y., Kaplan G.G., Ng S.C., Wilson D.C., Cameron F., Henderson P., Kotze P.G. (2022). Twenty-first Century Trends in the Global Epidemiology of Pediatric-Onset Inflammatory Bowel Disease: Systematic Review. Gastroenterology.

[3] Zhang Y.Z., Li Y.Y. (2014). Inflammatory bowel disease: Pathogenesis. World J. Gastroenterol..

[4] Nishida A., Inoue R., Inatomi O., Bamba S., Naito Y., Andoh A. (2018). Gut microbiota in the pathogenesis of inflammatory bowel disease. Clin. J. Gastroenterol..

[5] Kalla R., Ventham N.T., Kennedy N.A., Quintana J.F., Nimmo E.R., Buck A.H., Satsangi J. (2015). MicroRNAs: New players in IBD. Gut.

[6] Cai Y., Yu X., Hu S., Yu J. (2009). A brief review on the mechanisms of miRNA regulation. Genom. Proteom. Bioinform..

[7] Alamdari-Palangi V., Vahedi F., Shabaninejad Z., Dokeneheifard S., Movehedpour A., Taheri-Anganeh M., Savardashtaki A. (2021). microRNA in inflammatory bowel disease at a glance. Eur. J. Gastroenterol. Hepatol..

[8] Bocchetti M., Ferraro M.G., Ricciardiello F., Ottaiano A., Luce A., Cossu A.M., Scrima M., Leung W.-Y., Abate M., Stiuso P. (2021). The Role of microRNAs in Development of Colitis-Associated Colorectal Cancer. Int. J. Mol. Sci..

[9] Rogler G., Singh A., Kavanaugh A., Rubin D.T. (2021). Extraintestinal Manifestations of Inflammatory Bowel Disease: Current Concepts, Treatment, and Implications for Disease Management. Gastroenterology.

[10] Gao X., Tang Y., Lei N., Luo Y., Chen P., Liang C., Duan S., Zhang Y. (2021). Symptoms of anxiety/depression is associated with more aggressive inflammatory bowel disease. Sci. Rep..

[11] Du L., Ha C. (2020). Epidemiology and Pathogenesis of Ulcerative Colitis. Gastroenterol. Clin. N. Am..

[12] Wu F., Zikusoka M., Trindade A., Dassopoulos T., Harris M.L., Bayless T.M., Brant S.R., Chakravarti S., Kwon J.H. (2008). MicroRNAs are differentially expressed in ulcerative colitis and alter expression of macrophage inflammatory peptide-2 alpha. Gastroenterology.

[13] Guz M., Dworzański T., Jeleniewicz W., Cybulski M., Kozicka J., Stepulak A., Celiński K. (2020). Elevated miRNA Inversely Correlates with E-cadherin Gene Expression in Tissue Biopsies from Crohn Disease Patients in contrast to Ulcerative Colitis Patients. Biomed. Res. Int..

[14] Schaefer J.S., Attumi T., Opekun A.R., Abraham B., Hou J., Shelby H., Graham D.Y., Streckfus C., Klein J.R. (2015). MicroRNA signatures differentiate Crohn’s disease from ulcerative colitis. BMC Immunol..

[15] Wu F., Guo N.J., Tian H., Marohn M., Gearhart S., Bayless T.M., Brant S.R., Kwon J.H. (2011). Peripheral blood microRNAs distinguish active ulcerative colitis and Crohn’s disease. Inflamm. Bowel Dis..

[16] Netz U., Carter J., Eichenberger M.R., Feagins K., Galbraith N.J., Dryden G.W., Pan J., Rai S.N., Galandiuk S. (2017). Plasma microRNA Profile Differentiates Crohn’s Colitis From Ulcerative Colitis. Inflamm. Bowel Dis..

[17] Chen P., Li Y., Li L., Yu Q., Chao K., Zhou G., Qiu Y., Feng R., Huang S., He Y. (2019). Circulating microRNA146b-5p is superior to C-reactive protein as a novel biomarker for monitoring inflammatory bowel disease. Aliment. Pharmacol. Ther..

[18] Verdier J., Breunig I.R., Ohse M.C., Roubrocks S., Kleinfeld S., Roy S., Streetz K., Trautwein C., Roderburg C., Sellge G. (2020). Faecal Micro-RNAs in Inflammatory Bowel Diseases. J. Crohns Colitis.

[19] Schonauen K., Le N., von Arnim U., Schulz C., Malfertheiner P., Link A. (2018). Circulating and Fecal microRNAs as Biomarkers for Inflammatory Bowel Diseases. Inflamm. Bowel Dis..

[20] Ahmed F.E., Jeffries C.D., Vos P.W., Flake G., Nuovo G.J., Sinar D.R., Naziri W., Marcuard S.P. (2009). Diagnostic microRNA markers for screening sporadic human colon cancer and active ulcerative colitis in stool and tissue. Cancer Genom. Proteom..

[21] Lian H., Zhong X.S., Xiao Y., Sun Z., Shen Y., Zhao K., Ma X., Li Y., Niu Q., Liu M. (2022). Exosomal miR-29b of Gut Origin in Patients With Ulcerative Colitis Suppresses Heart Brain-Derived Neurotrophic Factor. Front. Mol. Biosci..

[22] Bayraktar R., Bertilaccio M.T.S., Calin G.A. (2019). The Interaction Between Two Worlds: MicroRNAs and Toll-Like Receptors. Front. Immunol..

[23] Guo X.Y., Liu X.J., Hao J.Y. (2020). Gut microbiota in ulcerative colitis: Insights on pathogenesis and treatment. J. Dig. Dis..

[24] Rhee S.H., Hwang D. (2000). Murine TOLL-like receptor 4 confers lipopolysaccharide responsiveness as determined by activation of NF kappa B and expression of the inducible cyclooxygenase. J. Biol. Chem..

[25] Ando Y., Mazzurana L., Forkel M., Okazaki K., Aoi M., Schmidt P.T., Mjösberg J., Bresso F. (2016). Downregulation of MicroRNA-21 in Colonic CD3+ T Cells in UC Remission. Inflamm. Bowel Dis..

[26] Gwiggner M., Martinez-Nunez R.T., Whiteoak S.R., Bondanese V.P., Claridge A., Collins J.E., Cummings J.R.F., Sanchez-Elsner T. (2018). MicroRNA-31 and MicroRNA-155 Are Overexpressed in Ulcerative Colitis and Regulate IL-13 Signaling by Targeting Interleukin 13 Receptor α-1. Genes.

[27] Heller F., Florian P., Bojarski C., Richter J., Christ M., Hillenbrand B., Mankertz J., Gitter A.H., Bürgel N., Fromm M. (2005). Interleukin-13 is the key effector Th2 cytokine in ulcerative colitis that affects epithelial tight junctions, apoptosis, and cell restitution. Gastroenterology.

[28] Heller F., Fromm A., Gitter A.H., Mankertz J., Schulzke J.D. (2008). Epithelial apoptosis is a prominent feature of the epithelial barrier disturbance in intestinal inflammation: Effect of pro-inflammatory interleukin-13 on epithelial cell function. Mucosal Immunol..

[29] Alam K.J., Mo J.S., Han S.H., Park W.C., Kim H.S., Yun K.J., Chae S.C. (2017). MicroRNA 375 regulates proliferation and migration of colon cancer cells by suppressing the CTGF-EGFR signaling pathway. Int. J. Cancer.

[30] Veauthier B., Hornecker J.R. (2018). Crohn’s Disease: Diagnosis and Management. Am. Fam. Physician.

[31] Rosen M.J., Dhawan A., Saeed S.A. (2015). Inflammatory Bowel Disease in Children and Adolescents. JAMA Pediatr..

[32] Wu F., Zhang S., Dassopoulos T., Harris M.L., Bayless T.M., Meltzer S.J., Brant S.R., Kwon J.H. (2010). Identification of microRNAs associated with ileal and colonic Crohn’s disease. Inflamm. Bowel Dis..

[33] Nijhuis A., Biancheri P., Lewis A., Bishop C.L., Giuffrida P., Chan C., Feakins R., Poulsom R., Di Sabatino A., Corazza G.R. (2014). In Crohn’s disease fibrosis-reduced expression of the miR-29 family enhances collagen expression in intestinal fibroblasts. Clin. Sci. (Lond.).

[34] Wohnhaas C.T., Schmid R., Rolser M., Kaaru E., Langgartner D., Rieber K., Strobel B., Eisele C., Wiech F., Jakob I. (2020). Fecal MicroRNAs Show Promise as Noninvasive Crohn’s Disease Biomarkers. Crohns Colitis 360.

[35] Chuang A.Y., Chuang J.C., Zhai Z., Wu F., Kwon J.H. (2014). NOD2 expression is regulated by microRNAs in colonic epithelial HCT116 cells. Inflamm. Bowel Dis..

[36] Brain O., Owens B.M., Pichulik T., Allan P., Khatamzas E., Leslie A., Steevels T., Sharma S., Mayer A., Catuneanu A.M. (2013). The intracellular sensor NOD2 induces microRNA-29 expression in human dendritic cells to limit IL-23 release. Immunity.

[37] Lewis A., Nijhuis A., Mehta S., Kumagai T., Feakins R., Lindsay J.O., Silver A. (2015). Intestinal fibrosis in Crohn’s disease: Role of microRNAs as fibrogenic modulators, serum biomarkers, and therapeutic targets. Inflamm. Bowel Dis..

[38] Boros É., Nagy I. (2019). The Role of MicroRNAs upon Epithelial-to-Mesenchymal Transition in Inflammatory Bowel Disease. Cells.

[39] M’Koma A.E., Moses H.L., Adunyah S.E. (2011). Inflammatory bowel disease-associated colorectal cancer: Proctocolectomy and mucosectomy do not necessarily eliminate pouch-related cancer incidences. Int. J. Colorectal Dis..

[40] Kara M., Yumrutas O., Ozcan O., Celik O.I., Bozgeyik E., Bozgeyik I., Tasdemir S. (2015). Differential expressions of cancer-associated genes and their regulatory miRNAs in colorectal carcinoma. Gene.

[41] Feng Y., Zhang Y., Zhou D., Chen G., Li N. (2019). MicroRNAs, intestinal inflammatory and tumor. Bioorg. Med. Chem. Lett..

[42] El-Daly S.M., Omara E.A., Hussein J., Youness E.R., El-Khayat Z. (2019). Differential expression of miRNAs regulating NF-κB and STAT3 crosstalk during colitis-associated tumorigenesis. Mol. Cell Probes.

[43] Proença M.A., Biselli J.M., Succi M., Severino F.E., Berardinelli G.N., Caetano A., Reis R.M., Hughes D.J., Silva A.E. (2018). Relationship between Fusobacterium nucleatum, inflammatory mediators and microRNAs in colorectal carcinogenesis. World J. Gastroenterol..

[44] Dai X., Xie Y., Dong M. (2022). Cancer-associated fibroblasts derived extracellular vesicles promote angiogenesis of colorectal adenocarcinoma cells through miR-135b-5p/FOXO1 axis. Cancer Biol. Ther..

[45] Lai C.Y., Yeh K.Y., Liu B.F., Chang T.M., Chang C.H., Liao Y.F., Liu Y.W., Her G.M. (2021). MicroRNA-21 Plays Multiple Oncometabolic Roles in Colitis-Associated Carcinoma and Colorectal Cancer via the PI3K/AKT, STAT3, and PDCD4/TNF-α Signaling Pathways in Zebrafish. Cancers.

[46] Guo J., Liao M., Wang J. (2021). TLR4 signaling in the development of colitis-associated cancer and its possible interplay with microRNA-155. Cell Commun. Signal..

[47] He C., Yu T., Shi Y., Ma C., Yang W., Fang L., Sun M., Wu W., Xiao F., Guo F. (2017). MicroRNA 301A Promotes Intestinal Inflammation and Colitis-Associated Cancer Development by Inhibiting BTG1. Gastroenterology.

[48] Wang T., Xu X., Xu Q., Ren J., Shen S., Fan C., Hou Y. (2017). miR-19a promotes colitis-associated colorectal cancer by regulating tumor necrosis factor alpha-induced protein 3-NF-κB feedback loops. Oncogene.

[49] Signs S.A., Fisher R.C., Tran U., Chakrabarti S., Sarvestani S.K., Xiang S., Liska D., Roche V., Lai W., Gittleman H.R. (2018). Stromal miR-20a controls paracrine CXCL8 secretion in colitis and colon cancer. Oncotarget.

[50] Butin-Israeli V., Bui T.M., Wiesolek H.L., Mascarenhas L., Lee J.J., Mehl L.C., Knutson K.R., Adam S.A., Goldman R.D., Beyder A. (2019). Neutrophil-induced genomic instability impedes resolution of inflammation and wound healing. J. Clin. Investig..

[51] Yang Y., Weng W., Peng J., Hong L., Yang L., Toiyama Y., Gao R., Liu M., Yin M., Pan C. (2017). Fusobacterium nucleatum Increases Proliferation of Colorectal Cancer Cells and Tumor Development in Mice by Activating Toll-Like Receptor 4 Signaling to Nuclear Factor-κB, and Up-regulating Expression of MicroRNA-21. Gastroenterology.

[52] Ma J., Yang Y., Fu Y., Guo F., Zhang X., Xiao S., Zhu W., Huang Z., Zhang J., Chen J. (2018). PIAS3-mediated feedback loops promote chronic colitis-associated malignant transformation. Theranostics.

[53] Liu S., Sun X., Wang M., Hou Y., Zhan Y., Jiang Y., Liu Z., Cao X., Chen P., Chen X. (2014). A microRNA 221- and 222-mediated feedback loop maintains constitutive activation of NFκB and STAT3 in colorectal cancer cells. Gastroenterology.

[54] Zhu Y., Gu L., Li Y., Lin X., Shen H., Cui K., Chen L., Zhou F., Zhao Q., Zhang J. (2017). miR-148a inhibits colitis and colitis-associated tumorigenesis in mice. Cell Death Differ..

[55] Tang K., Wu Z., Sun M., Huang X., Sun J., Shi J., Wang X., Miao Z., Gao P., Song Y. (2022). Elevated MMP10/13 mediated barrier disruption and NF-κB activation aggravate colitis and colon tumorigenesis in both individual or full miR-148/152 family knockout mice. Cancer Lett..

[56] Dougherty U., Mustafi R., Zhu H., Zhu X., Deb D., Meredith S.C., Ayaloglu-Butun F., Fletcher M., Sanchez A., Pekow J. (2021). Upregulation of polycistronic microRNA-143 and microRNA-145 in colonocytes suppresses colitis and inflammation-associated colon cancer. Epigenetics.

[57] Lamichhane S., Mo J.S., Sharma G., Choi T.Y., Chae S.C. (2021). MicroRNA 452 regulates IL20RA-mediated JAK1/STAT3 pathway in inflammatory colitis and colorectal cancer. Inflamm. Res..

[58] Zhang W., Fu X., Xie J., Pan H., Han W., Huang W. (2021). miR-26a attenuates colitis and colitis-associated cancer by targeting the multiple intestinal inflammatory pathways. Mol. Ther. Nucleic Acids.

[59] Chen C.Y., Chang J.T., Ho Y.F., Shyu A.B. (2016). MiR-26 down-regulates TNF-α/NF-κB signalling and IL-6 expression by silencing HMGA1 and MALT1. Nucleic Acids Res..

[60] Zhou R., Qiu P., Wang H., Yang H., Yang X., Ye M., Wang F., Zhao Q. (2021). Identification of microRNA-16-5p and microRNA-21-5p in feces as potential noninvasive biomarkers for inflammatory bowel disease. Aging (Albany N. Y.).

[61] Schicho R., Marsche G., Storr M. (2015). Cardiovascular complications in inflammatory bowel disease. Curr. Drug Targets.

[62] Rungoe C., Nyboe Andersen N., Jess T. (2015). Inflammatory bowel disease and risk of coronary heart disease. Trends Cardiovasc Med..

[63] Vikram A., Kim Y.R., Kumar S., Li Q., Kassan M., Jacobs J.S., Irani K. (2016). Vascular microRNA-204 is remotely governed by the microbiome and impairs endothelium-dependent vasorelaxation by downregulating Sirtuin1. Nat. Commun..

[64] Kassan A., Ait-Aissa K., Kassan M. (2021). Hypothalamic miR-204 Induces Alteration of Heart Electrophysiology and Neurogenic Hypertension by Regulating the Sympathetic Nerve Activity: Potential Role of Microbiota. Cureus.

[65] Tang Y., Kline K.T., Zhong X.S., Xiao Y., Lian H., Peng J., Liu X., Powell D.W., Tang G., Li Q. (2021). Chronic colitis upregulates microRNAs suppressing brain-derived neurotrophic factor in the adult heart. PLoS ONE.

[66] Pius-Sadowska E., Machaliński B. (2017). BDNF—A key player in cardiovascular system. J. Mol. Cell. Cardiol..

[67] Do J., Woo J. (2018). From Gut to Brain: Alteration in Inflammation Markers in the Brain of Dextran Sodium Sulfate-induced Colitis Model Mice. Clin. Psychopharmacol. Neurosci..

[68] Mayer E.A., Nance K., Chen S. (2022). The Gut-Brain Axis. Annu. Rev. Med..

[69] Rosa J.M., Formolo D.A., Yu J., Lee T.H., Yau S.Y. (2022). The Role of MicroRNA and Microbiota in Depression and Anxiety. Front. Behav. Neurosci..

[70] Dinan T.G., Cryan J.F. (2017). The Microbiome-Gut-Brain Axis in Health and Disease. Gastroenterol. Clin. N. Am..

[71] Jang H.M., Kim J.K., Joo M.K., Shin Y.J., Lee C.K., Kim H.J., Kim D.H. (2021). Transplantation of fecal microbiota from patients with inflammatory bowel disease and depression alters immune response and behavior in recipient mice. Sci. Rep..

[72] Numakawa T., Richards M., Adachi N., Kishi S., Kunugi H., Hashido K. (2011). MicroRNA function and neurotrophin BDNF. Neurochem. Int..

[73] Al-Qudah M., Shammala D.A., Al-Dwairi A., Al-Shboul O., Mustafa A.G. (2017). Dextran sodium sulphate (DSS)-induced colitis alters the expression of neurotrophins in smooth muscle cells of rat colon. Physiol. Res..

[74] Huan Z., Mei Z., Na H., Xinxin M., Yaping W., Ling L., Lei W., Kejin Z., Yanan L. (2021). lncRNA MIR155HG Alleviates Depression-Like Behaviors in Mice by Regulating the miR-155/BDNF Axis. Neurochem. Res..

[75] Yang W., Liu M., Zhang Q., Zhang J., Chen J., Chen Q., Suo L. (2020). Knockdown of miR-124 Reduces Depression-like Behavior by Targeting CREB1 and BDNF. Curr. Neurovasc. Res..

[76] Malik T.F., Aurelio D.M. (2022). Extraintestinal Manifestations of Inflammatory Bowel Disease. StatPearls.

[77] Lu Y., Cao D.L., Zhao L.X., Han Y., Zhang Y.L. (2018). MicroRNA-146a-5p attenuates visceral hypersensitivity through targeting chemokine CCL8 in the spinal cord in a mouse model of colitis. Brain Res. Bull..

[78] Greuter T., Vavricka S.R. (2019). Extraintestinal manifestations in inflammatory bowel disease—Epidemiology, genetics, and pathogenesis. Expert Rev. Gastroenterol. Hepatol..

[79] Cordes F., Demmig C., Bokemeyer A., Brückner M., Lenze F., Lenz P., Nowacki T., Tepasse P., Schmidt H.H., Schmidt M.A. (2020). MicroRNA-320a Monitors Intestinal Disease Activity in Patients With Inflammatory Bowel Disease. Clin. Transl. Gastroenterol..

[80] James J.P., Riis L.B., Malham M., Høgdall E., Langholz E., Nielsen B.S. (2020). MicroRNA Biomarkers in IBD-Differential Diagnosis and Prediction of Colitis-Associated Cancer. Int. J. Mol. Sci..

[81] Vivinus-Nébot M., Frin-Mathy G., Bzioueche H., Dainese R., Bernard G., Anty R., Filippi J., Saint-Paul M.C., Tulic M.K., Verhasselt V. (2014). Functional bowel symptoms in quiescent inflammatory bowel diseases: Role of epithelial barrier disruption and low-grade inflammation. Gut.

[82] Jelsness-Jørgensen L.P., Bernklev T., Moum B. (2013). Calprotectin Is a Useful Tool in Distinguishing Coexisting Irritable Bowel-Like Symptoms from That of Occult Inflammation among Inflammatory Bowel Disease Patients in Remission. Gastroenterol. Res. Pract..

[83] Keohane J., O’Mahony C., O’Mahony L., O’Mahony S., Quigley E.M., Shanahan F. (2010). Irritable bowel syndrome-type symptoms in patients with inflammatory bowel disease: A real association or reflection of occult inflammation?. Am. J. Gastroenterol..

[84] Williams A.D., Korolkova O.Y., Sakwe A.M., Geiger T.M., James S.D., Muldoon R.L., Herline A.J., Goodwin J.S., Izban M.G., Washington M.K. (2017). Human alpha defensin 5 is a candidate biomarker to delineate inflammatory bowel disease. PLoS ONE.

[85] Cordes F., Brückner M., Lenz P., Veltman K., Glauben R., Siegmund B., Hengst K., Schmidt M.A., Cichon C., Bettenworth D. (2016). MicroRNA-320a Strengthens Intestinal Barrier Function and Follows the Course of Experimental Colitis. Inflamm. Bowel Dis..

[86] Zhou Q., Costinean S., Croce C.M., Brasier A.R., Merwat S., Larson S.A., Basra S., Verne G.N. (2015). MicroRNA 29 targets nuclear factor-κB-repressing factor and Claudin 1 to increase intestinal permeability. Gastroenterology.

[87] Mahurkar-Joshi S., Rankin C.R., Videlock E.J., Soroosh A., Verma A., Khandadash A., Iliopoulos D., Pothoulakis C., Mayer E.A., Chang L. (2021). The Colonic Mucosal MicroRNAs, MicroRNA-219a-5p, and MicroRNA-338-3p Are Downregulated in Irritable Bowel Syndrome and Are Associated With Barrier Function and MAPK Signaling. Gastroenterology.

[88] Chira A., Muresan M.S., Braicu C., Budisan L., Raduly L., Chira R.I., Dumitrascu D.L., Berindan-Neagoe I. (2020). Serum patterns of mir-23a and mir-181b in irritable bowel syndrome and colorectal cancer—A pilot study. Bosn. J. Basic Med. Sci..

[89] Fourie N.H., Peace R.M., Abey S.K., Sherwin L.B., Rahim-Williams B., Smyser P.A., Wiley J.W., Henderson W.A. (2014). Elevated circulating miR-150 and miR-342-3p in patients with irritable bowel syndrome. Exp. Mol. Pathol..

[90] Mansour M.A., Sabbah N.A., Mansour S.A., Ibrahim A.M. (2016). MicroRNA-199b expression level and coliform count in irritable bowel syndrome. IUBMB Life.

[91] Ahmed Hassan E., El-Din Abd El-Rehim A.S., Mohammed Kholef E.F., Abd-Elgwad Elsewify W. (2020). Potential role of plasma miR-21 and miR-92a in distinguishing between irritable bowel syndrome, ulcerative colitis, and colorectal cancer. Gastroenterol. Hepatol. Bed Bench.

[92] Ye J., Xu M., Tian X., Cai S., Zeng S. (2019). Research advances in the detection of miRNA. J. Pharm. Anal..

[93] Dave V.P., Ngo T.A., Pernestig A.K., Tilevik D., Kant K., Nguyen T., Wolff A., Bang D.D. (2019). MicroRNA amplification and detection technologies: Opportunities and challenges for point of care diagnostics. Lab. Invest..

[94] Casado-Bedmar M., Viennois E. (2021). microRNA and gut microbiota: Tiny but mighty—Novel insights of their crosstalk in inflammatory bowel disease pathogenesis and therapeutics. J. Crohns Colitis.

[95] Coskun M., Bjerrum J.T., Seidelin J.B., Troelsen J.T., Olsen J., Nielsen O.H. (2013). miR-20b, miR-98, miR-125b-1*, and let-7e* as new potential diagnostic biomarkers in ulcerative colitis. World J. Gastroenterol..

[96] Soroosh A., Koutsioumpa M., Pothoulakis C., Iliopoulos D. (2018). Functional role and therapeutic targeting of microRNAs in inflammatory bowel disease. Am. J. Physiol. Gastrointest. Liver Physiol..

[97] Suri K., Bubier J.A., Wiles M.V., Shultz L.D., Amiji M.M., Hosur V. (2021). Role of MicroRNA in Inflammatory Bowel Disease: Clinical Evidence and the Development of Preclinical Animal Models. Cells.

[98] Polytarchou C., Hommes D.W., Palumbo T., Hatziapostolou M., Koutsioumpa M., Koukos G., van der Meulen-de Jong A.E., Oikonomopoulos A., van Deen W.K., Vorvis C. (2015). MicroRNA214 Is Associated With Progression of Ulcerative Colitis, and Inhibition Reduces Development of Colitis and Colitis-Associated Cancer in Mice. Gastroenterology.

[99] Yu Y., Liao L., Shao B., Su X., Shuai Y., Wang H., Shang F., Zhou Z., Yang D., Jin Y. (2017). Knockdown of MicroRNA Let-7a Improves the Functionality of Bone Marrow-Derived Mesenchymal Stem Cells in Immunotherapy. Mol. Ther..

[100] Luo J., Wang Y., Lan D., Niu J., Miao J., Dong X., Yang G., Zhang F., Cao Y., Wang K. (2018). Differential expression of serum microRNAs in glucocorticoid-resistant patients with ulcerative colitis. Int. J. Clin. Exp. Pathol..

